# Diagnostic Accuracy of SARS-CoV-2 Antigen Tests for Community Transmission Screening: A Systematic Review and Meta-Analysis

**DOI:** 10.3390/ijerph182111451

**Published:** 2021-10-30

**Authors:** Cheng-Chieh Chen, Shou-Cheng Lu, Chyi-Huey Bai, Pei-Yu Wang, Kang-Yun Lee, Yuan-Hung Wang

**Affiliations:** 1 Graduate Institute of Clinical Medicine, College of Medicine, Taipei Medical University, Taipei 11031, Taiwan; jack10231024@gmail.com; 2 Department of Pathology and Laboratory Medicine, Shin Kong Wu Ho-Su Memorial Hospital, Taipei 11101, Taiwan; 3 Department of Laboratory Medicine, Shuang Ho Hospital, Taipei Medical University, New Taipei City 23561, Taiwan; 08395@s.tmu.edu.tw (S.-C.L.); 16127@s.tmu.edu.tw (P.-Y.W.); 4 Department of Public Health, College of Medicine, Taipei Medical University, Taipei 11031, Taiwan; baich@ms4.hinet.net; 5 Division of Pulmonary Medicine, Department of Internal Medicine, Shuang Ho Hospital, Taipei Medical University, New Taipei City 23561, Taiwan; leekangyun@tmu.edu.tw; 6 Division of Pulmonary Medicine, Department of Internal Medicine, School of Medicine, College of Medicine, Taipei Medical University, Taipei 11031, Taiwan; 7 Department of Medical Research, Shuang Ho Hospital, Taipei Medical University, New Taipei City 23561, Taiwan

**Keywords:** antigen test, COVID-19, meta-analysis, SARS-CoV-2, sensitivity and specificity

## Abstract

Severe Acute Respiratory Syndrome Coronavirus-2 (SARS-CoV-2) caused the global pandemic of coronavirus disease 2019 (COVID-19). Rapid identification and isolation of infectious patients are critical methods to block COVID-19 transmission. Antigen tests can contribute to prompt identification of infectious individuals. This meta-analysis aims to evaluate the diagnostic accuracy of antigen tests for SARS-CoV-2. We conducted a literature search in PubMed, Embase, the Cochrane Library, and Biomed Central databases. Studies evaluating the diagnostic accuracy of antigen tests for SARS-CoV-2 in community participants were included. Only English-language articles were reviewed. We included eligible studies that provided available data to construct a 2 × 2 table on a per-patient basis. Overall sensitivity and specificity for antigen tests were generated using a bivariate random-effects model. Eighteen studies with 34,865 participants were retrieved. The meta-analysis for SARS-CoV-2 antigen tests generated a pooled sensitivity of 0.82 and a pooled specificity of 1.00. A subgroup analysis of ten studies that reported outcomes for 5629 symptomatic participants generated a pooled sensitivity of 0.87 and a pooled specificity of 1.00. Antigen tests might have higher sensitivity in detecting SARS-CoV-2 in symptomatic patients in the community and may be an effective tool to identify patients to be quarantined to prevent further SARS-CoV-2 transmission.

## 1. Introduction

Severe Acute Respiratory Syndrome Coronavirus-2 (SARS-CoV-2) caused the global pandemic of coronavirus disease 2019 (COVID-19). Asymptomatic cases make COVID-19 difficult to monitor and prevent. It is estimated that at least 50% of COVID-19 patients contract the virus from asymptomatic people [1]. To break the transmission chains of SARS-CoV-2, testing infected individuals and tracing and quarantining their contacts have been used as major nonpharmaceutical interventions [2]. Rapid identification and isolation of infectious patients with SARS-CoV-2 are critical methods to block COVID-19 community transmission. Approximately 40% of infected individuals with high viral loads might be asymptomatic [3]. The World Health Organization and Centers for Disease Control and Prevention have implemented reverse-transcription polymerase chain reaction (RT-PCR) technology as the standard diagnostic assay for SARS-CoV-2 detection. RT-PCR has a high sensitivity for SARS-CoV-2. The sensitivity of RT-PCR ranged from 71 to 98%, and the assay was 100% specific [4,5]. However, factors such as the type and quality of the respiratory specimen and the stage of the disease influence testing accuracy. Despite its high sensitivity, RT-PCR has disadvantages, including the necessity of professional lab expertise, costly reagents, and centralized equipment. Therefore, antigen tests that detect viral proteins of SARS-CoV-2 in respiratory samples have been developed [6]. Antigen tests are relatively inexpensive, and most of them can be used at the point of care. Antigen tests can identify individuals with COVID-19 who are highly contagious, namely those whose viral load is likely to be high. Antigen tests have received the U.S. Food and Drug Administration Emergency Use Authorization for use in asymptomatic and symptomatic individuals [7].

The advantages of antigen tests, such as relatively low cost and short turnaround time, can contribute to prompt identification of infectious individuals. RT-PCR testing should be considered after negative antigen test results in symptomatic individuals and after positive antigen test results in asymptomatic individuals [8]. Although antigen tests might not be as accurate as RT-PCR testing, they are more accessible in terms of availability and ease of use and can be used to scale up testing outside of laboratory settings (e.g., frequent repeat testing) [9]. A crucial role for testing in the COVID-19 pandemic response is in identifying people who are not infected with SARS-CoV-2 so that they can travel, return to school or work, and attend mass gatherings. The wide availability of antigen tests and their rapid turnaround time offer the promise of efficiently testing a large number of people in the community [9].

The diagnostic accuracy of using antigen tests for COVID-19 among members of the community at large is still inconclusive. Therefore, the aim of this meta-analysis was to evaluate the accuracy of antigen tests for detecting SARS-CoV-2 among suspected COVID-19 patients in the community.

## 2. Materials and Methods

### 2.1. Literature Search Strategy

The study was reported according to “Preferred Reporting Items for a Systematic Review and Meta-Analysis of Diagnostic Test Accuracy Studies: The PRISMA-DTA Statement” [10].

We conducted a literature search for relevant studies in PubMed, Embase, the Cochrane Library, and Biomed Central. A literature search was conducted using multiple search terms, including (COVID-19 OR severe acute respiratory syndrome coronavirus 2 OR SARS-CoV-2) AND (antigen test OR SARS-CoV-2 antigens OR Mass Screening OR Community Participation) AND (RT-PCR OR Reverse Transcriptase Polymerase Chain Reaction OR COVID-19 Nucleic Acid Testing) AND (sensitivity OR specificity). A combination of free text and MeSH terms was used to identify relevant studies. We limited our search results to studies performed with human participants. Detailed search strategies are presented in Table S1.

### 2.2. Inclusion and Exclusion Criteria

Studies evaluating the diagnostic accuracy of antigen tests for SARS-CoV-2 with reference standards in participants with suspected SARS-CoV-2 infection in the community were included, but review articles were excluded. Respiratory specimens were collected from symptomatic or asymptomatic individuals. Studies that defined RT-PCR technology as the reference standard were included. Only English-language articles were reviewed. The literature search was conducted with no time restrictions. Studies that provided sufficient data to construct a 2 × 2 table on a per-patient basis were included. We excluded case reports, case series, proposals, protocols, conference abstracts, in-house tests, and preprint articles. The last literature search was performed on 1 August 2021. One reviewer initially screened titles and abstracts for potentially eligible studies. After eliminating irrelevant studies, two reviewers independently examined full-text articles that met the inclusion criteria. Disagreements between the reviewers were resolved through joint discussions.

### 2.3. Quality Assessment

The quality of the included studies was assessed using the Quality Assessment of Diagnostic Accuracy Studies-2 (QUADAS-2) tool [11]. Antigen tests for the SARS-CoV-2 virus were the index tests and RT-PCR test results for SARS-CoV-2 were the reference standards. The QUADAS-2 tool consists of the following four domains: patient selection, index test, reference standard, and flow and timing. Each domain includes questions that allow an assessment of the risk of bias. The quality assessment of the diagnostic test comprises the risk of bias and the applicability for individual studies. A study is considered high-quality if each domain in the study exhibits a low risk of bias.

### 2.4. Statistical Analysis

We extracted data on true positives, true negatives, false positives, and false negatives from each included study to construct 2 × 2 tables for calculating values of the pooled sensitivity, pooled specificity. If 2 × 2 tables could not be extracted from the main text, we searched the supplementary material of the study for additional information. The sensitivity of a test is the proportion of those with the target condition correctly identified as having the condition, whereas the specificity of a test is the proportion of those without the target condition correctly identified as not having the condition [12].

We conducted a meta-analysis using a bivariate random-effects model to generate a summary of sensitivity, specificity on a per-patient basis. We also graphed the summary receiver operating characteristic (SROC) curve to determine the overall diagnostic performance of the index tests. The closer the curve approaches the upper-left corner, the higher the overall performance is [13]. Possible causes of heterogeneity between studies were explored through pre-specified subgroup analysis, which included the following: days after symptom onset, asymptomatic participants, and symptomatic individuals. Summary estimates, including pooled sensitivity, specificity, and DOR, were generated with associated 95% confidence intervals (CIs). All analyses were performed using MetaDiSc version 1.4 (Universidad Complutense, Madrid, Spain) and MetaDTA software (National Institute for Health Research Complex Review Support Unit, Glasgow, UK) [14,15]. A value of *p* < 0.05 was considered statistically significant.

## 3. Results

**Figure 1:**
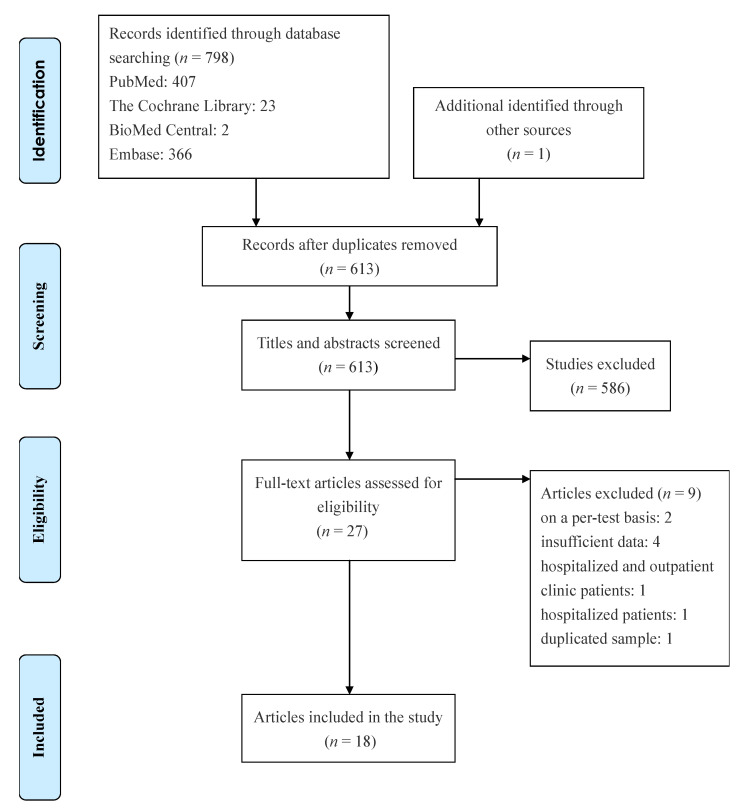
Flowchart of literature search.

**Figure 2:**
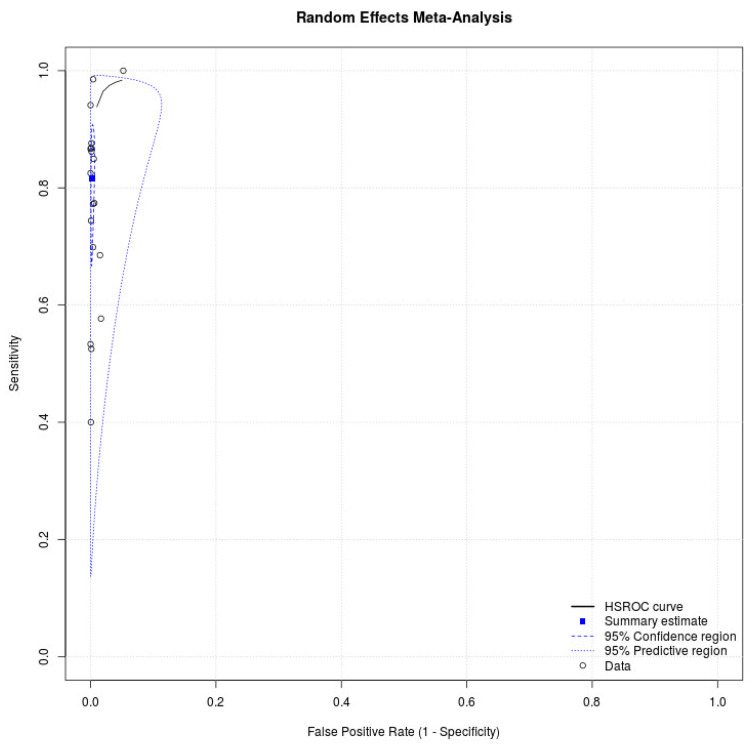
SROC curve showing the pooled sensitivity and specificity of antigen test for SARS-CoV-2.

Eighteen studies with 34,865 participants were retrieved [16,17,18,19,20,21,22,23,24,25,26,27,28,29,30,31,32,33]. Figure 1 depicts the process of the literature search, and Table 1 presents detailed characteristics of the studies. All studies in the meta-analysis used a prospective study design, and five studies enrolled participants in the drive-through testing sites [16,19,26,28,31]. Eight studies evaluated the diagnostic performance of antigen tests with nasal swab specimens [16,18,22,23,28,29,30,33], six assessed the accuracy of antigen tests with nasopharyngeal swab specimens [21,24,25,26,27,31], seven provided cycle threshold (Ct) values of positive RT-PCR tests [24,25,28,29,30,31,32], and eight reported cutoff values of Ct [16,21,22,23,25,27,28,33]. Table 2 lists the statistical data. The meta-analysis for antigen tests generated a pooled sensitivity of 0.82 (95% CI: 0.71–0.89) and a pooled specificity of 1.00 (95% CI: 0.99–1.00) (Figure 2). Eight studies with 16,470 patients discussed the accuracy of antigen tests using nasal swab specimens [16,18,22,23,28,29,30,33]. The meta-analysis produced a pooled sensitivity of 0.76 (95% CI: 0.58–0.88) and a pooled specificity of 1.00 (95% CI: 0.99–1.00). Moreover, six studies with 7441 patients reported the accuracy of antigen tests using nasopharyngeal swab specimens [21,24,25,26,27,31]. The meta-analysis produced a pooled sensitivity of 0.90 (95% CI: 0.76–0.96) and a pooled specificity of 1.00 (95% CI: 0.99–1.00). The supplementary information presents the sensitivities and specificities of antigen tests for SARS-CoV-2 from the included studies (see Figure S1).

### 3.1. Quality Assessment

**Figure 3:**
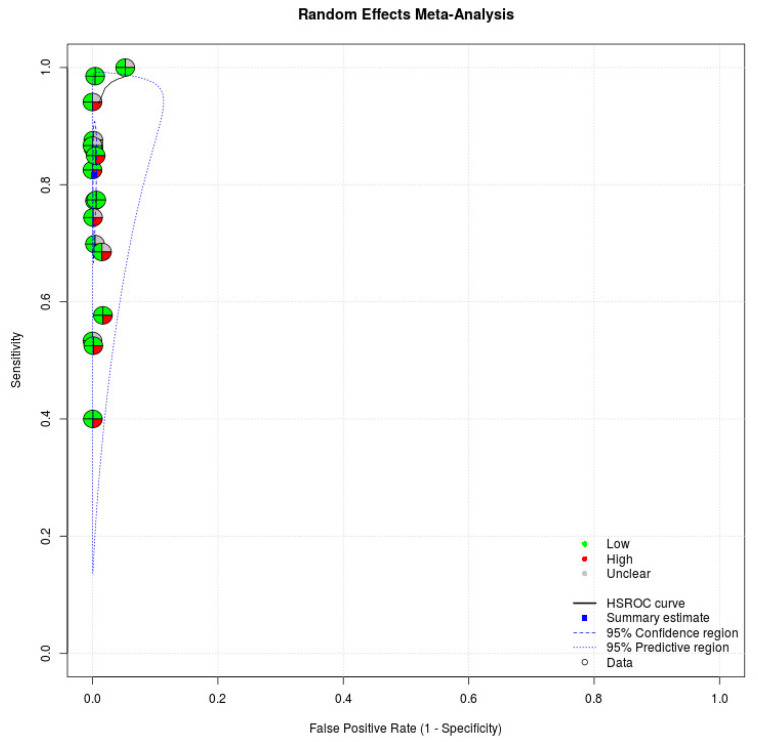
Risk of bias of individual studies. Circles of the SROC plot in MetaDTA are displayed as pie charts summarizing the risk of bias of individual studies based on the QUADAS-2 tool. The first quadrant of a circle represents patient selection, the second quadrant represents the index test, the third quadrant represents the reference standard, and the fourth quadrant represents flow and timing. Circles on the SROC plot are colored depending on their quality assessment score: green for low, red for high, and gray for unclear risk of bias.

We applied the QUADAS-2, which has four domains to evaluate the quality of studies, in our meta-analysis. Regarding patient selection, eight studies enrolled patients randomly or consecutively. All studies avoided a case–control study design, which might have overestimated the diagnostic accuracy. Based on the rules in this domain, eight studies were judged to have a low risk of bias in the patient selection domain [16,17,18,19,28,30,31,33]. Regarding index tests, all studies recorded that index tests were interpreted without knowledge of the results of the reference standard. All studies in the meta-analysis were judged to have a low risk of bias in the index domain. Regarding the reference standard, all studies indicated that the reference standard likely correctly classified the target condition. Regarding the flow and timing domain, 17 studies demonstrated that all patients received a reference standard [16,17,18,19,20,21,22,23,24,25,26,27,28,29,30,32,33]. Seven studies indicated that all patients were included in the analysis [24,25,26,27,28,30,33]. Five articles were judged as having a low risk of bias in the flow and timing domain [24,26,27,28,33]. With regard to applicability, index tests and reference standards of studies in our meta-analysis matched our review title. Table 3 presents the quality of studies. Figure 3 demonstrates the risk of bias of individual studies in the meta-analysis.

### 3.2. Investigation of Heterogeneity

Symptoms and the duration from symptom onset to specimen collection could represent sources of heterogeneity in the meta-analysis. We performed subgroup analyses to identify sources of heterogeneity. The *I*^2^ index represents heterogeneity across studies, with values of 25%, 50%, and 75% representing low, moderate, and high levels of heterogeneity, respectively [34]. According to the data of antigen tests for symptomatic patients, we performed a subgroup analysis for ten studies that reported outcomes for 5629 symptomatic participants [16,18,19,20,22,23,26,28,30,33]. This analysis generated a pooled sensitivity of 0.87 (95% CI: 0.78–0.93; *I*^2^ = 91.0%) and a pooled specificity of 1.00 (95% CI: 0.99–1.00; *I*^2^ = 81.7%). This indicates that antigen tests might have high sensitivity in the detection of COVID-19 among symptomatic participants. The subgroup analysis for nine studies that included 16,733 asymptomatic participants generated a pooled sensitivity of 0.57 (95% CI: 0.47–0.66; *I*^2^ = 85.0%) and a pooled specificity of 1.00 (95% CI: 0.99–1.00; *I*^2^ = 90.4%), respectively [16,17,19,21,22,23,28,29,30]. Based on the data of antigen tests for patients within 7 days after symptom onset, we performed another subgroup analysis. Three studies with 2046 patients in the meta-analysis reported data of antigen tests for participants within 7 days after symptom onset [22,28,33]. The subgroup analysis indicated a pooled sensitivity of 0.97 (95% CI: 0.69–1.00; *I*^2^ = 95.2%) and a pooled specificity of 1.00 (95% CI: 0.99–1.00; *I*^2^ = 63.3%), indicating that antigen tests might have higher pooled sensitivity in detecting SARS-CoV-2 in symptomatic patients with no more than 7 days of disease evolution. Five studies with 13,236 patients reported data of antigen tests using Ct cutoff value less than or equal to 35 [17,22,25,27,33]. The subgroup analysis indicated a pooled sensitivity of 0.93 (95% CI: 0.60–0.99; *I*^2^ = 97.3%) and a pooled specificity of 1.00 (95% CI: 0.98–1.00; *I*^2^ = 98.5%), indicating that antigen tests might have higher pooled sensitivity in detecting SARS-CoV-2 using a Ct cutoff value of 35. The supplementary information presents the statistical data of the subgroup analyses (see Table S2).

## 4. Discussion

Our major findings indicated that antigen tests had high sensitivity and excellent specificity in detecting SARS-CoV-2 in individuals in the community. If a test (in this case, an antigen test) has high specificity and yields a positive result, a clinician can be nearly certain that the disease (in this case, COVID-19) is present [35]. Antibody testing also plays a crucial role in understanding the seroprevalence of COVID-19 in the community and identifying individuals who are immunoreactive against SARS-CoV-2 [34]. RT-PCR is the standard diagnostic tool for SARS-CoV-2 detection. A previous study reported that RT-PCR positivity may persist over 3 weeks after illness onset, although most mild cases will yield a negative result. However, a positive RT-PCR result reveals only SARS-CoV-2 RNA and does not necessarily indicate the presence of a replicating virus [36].

Based on the subgroup analysis of our meta-analysis, antigen tests might have higher sensitivity in detecting SARS-CoV-2 in symptomatic individuals in the community, which indicates that antigen tests might be reliable for SARS-CoV-2 detection among the contagious population. In another subgroup analysis of studies involving asymptomatic participants, antigen tests had insufficient sensitivity in detecting SARS-CoV-2 in the asymptomatic population in the community. Surveillance testing regimens that can sever enough transmission chains to reduce community spread should complement current clinical diagnostic tests. Antigen tests could be used to enable true community-wide surveillance regimens for SARS-CoV-2 [37]. The current meta-analysis provided evidence of high sensitivity of antigen tests in identifying symptomatic individuals in the community. The antigen test is a valuable nonpharmaceutical intervention strategy to contain SARS-CoV-2. Recent research has suggested that when antigen tests are used, a pretest quarantine period of 5 days is not inferior to a quarantine of 10 days for travelers and a postexposure quarantine period of 10 days is not inferior to a quarantine of 14 days [38]. Gradual release of nonpharmaceutical interventions coupled with a high-efficacy vaccine strategy might prevent subsequent waves of SARS-CoV-2 transmission. Although vaccination can allow for some relaxation of nonpharmaceutical control measures, such relaxation should be performed gradually to avoid large-scale public health consequences [39].

Frequent use of antigen tests might help to identify infected individuals and reduce COVID-19 transmission [6] The benefits of administering antigen tests in suspected cases are the rapid diagnosis for clinical treatment and management (including protection of first-line staff) and the ability to quarantine infected individuals. Contact tracing becomes feasible so that positive cases can be isolated to minimize SARS-CoV-2 spread [40]. Diagnostic testing plays a key role in COVID-19 outbreak control. To end the pandemic, the accurate application of high-volume diagnostic testing and the rapid use of the results may help with the timely implementation of appropriate therapies and prevention of further spread [41]. Antigen tests could increase overall COVID-19 testing capacity and have the advantages of shorter turnaround times and reduced costs [42]. Antigen tests are most likely to have high performance in patients with high viral loads (Ct values ≤ 25), which usually appear in the presymptomatic (1–3 days before symptom onset) and early symptomatic (within the first 5–7 days of illness) phases of COVID-19 [43].

A study reported that a requirement to quarantine until an RT-PCR or antigen test on Day 7 after exposure (with early release if negative) might prevent as much transmission as the standard 14-day quarantine period [44]. Testing of asymptomatic health care workers has been suggested to reduce nosocomial transmission of COVID-19 [45]. Therefore, antigen tests can be used for screening and serial testing (every 2–3 days) of residents and staff in health care, home care, and long-term care facilities in areas where there is ongoing community transmission. When a first case is confirmed in a resident or staff member of a closed setting, a comprehensive testing strategy of all residents and staff should be considered [42].

Different testing strategies, including focused symptomatic testing, focused asymptomatic testing, mass testing, and systematic meaningful asymptomatic repeated testing, are adopted to prevent transmission. Many of these strategies use antigen tests [46]. Widespread community transmission has become entrenched in many countries and has required the testing of populations to identify and isolate infected individuals. Although the effects of mass antigen testing are difficult to distinguish from those of concurrent interventions, an obvious reduction in SARS-CoV-2 infections was observed after mass antigen testing in Slovakia. However, this reduction was restricted to regions with high SARS-CoV-2 prevalence, and testing had little effect in areas with lower viral prevalence [47]. Due to an increasing prevalence of new SARS-CoV2 variants with possible clinical implications, monitoring and detecting the spread of different variants in the general population in a timely method is critical [48]. Antigen testing is evolving. Sensitivity of SARS-the CoV-2 antigen test with a self-collected nasal swab is comparable with that of a professional-collected nasopharyngeal swab. Patients suspected of COVID-19 may be able to perform the antigen test and test by themselves [18]. Based on the outcomes of the meta-analysis, antigen tests might have higher sensitivity in symptomatic patients. Hence, we suggested that RT-PCR could be performed after negative antigen test results in symptomatic patients and positive antigen test results in asymptomatic patients in the community transmission screening algorithm for SARS-CoV-2 [8].

Although this meta-analysis demonstrated that antigen tests may be sensitive in detecting SARS-CoV-2 in the community, our study had limitations. The Ct cutoff values of the studies in the meta-analysis were limited. Statistical data of antigen tests stratified by Ct cutoff value are limited. Statistical data of antigen tests for patients under 18 years of age are limited. The majority of studies in this meta-analysis did not report the days after symptom onset of participants. No study in the meta-analysis reported SARS-CoV-2 variants.

## 5. Conclusions

Our major findings indicated that antigen tests had high sensitivity in detecting the SARS-CoV-2 virus in symptomatic patients in the community. Antigen tests might have a higher sensitivity in detecting SARS-CoV-2 within 7 days after symptom onset. Antigen tests are sensitive in detecting SARS-CoV-2 in patients with a Ct value less than or equal to 35. Therefore, antigen tests might be an effective tool in the effort to block SARS-CoV-2 transmission.

## Figures and Tables

**Table 1:** Characteristics of studies.

Study	Study Design	Testing Site	Patient Population	Prevalence (%)	Participants (Total/Data Extraction)	Age Median (Range)	Days After Symptom Onset Median (Range)	Specimen Type	Index Tests	Reference Standard	Ct Value of Positive RT-PCR Median (Range)	Threshold Value (Ct)
Pollock NR [16] 2021	Prospective	Drive-through testing site	Asymptomatic and symptomatic	15.6	(1063/1498)	NA	adult: 3 (0–44) children: 3 (1–20)	Anterior nasal swab	Access Bio CareStart COVID-19 Antigen test	RT-PCR	NA	≤25, ≤30, ≤35
García-Fiñana M [17] 2021	Prospective, cross sectional, consecutive	Community testing sites	Asymptomatic	1.3	(5869/5504)	50 (mean) (±18, SD)	NA	Self-administered swabs (combined throat and nose)	Innova lateral flow test	RT-PCR	NA	NA
Lindner AK [18] 2021	Prospective, consecutive	Ambulatory SARS-CoV-2 testing facility	Symptomatic	NA	(168/144)	35 (mean) (±11.5, SD)	3.4 (mean) (±2.0, SD)	Nasal swab (self-sampling)	STANDARD Q COVID-19 Ag Test	RT-PCR	NA	NA
Krüger LJ [19] 2021	Prospective	Drive-in testing site, clinical ambulatory testing facility	Asymptomatic and symptomatic	NA	(1261/1108)	39.4 (mean) (±14.1, SD)	4.01 (mean) (±3.1, SD)	Nasopharyngeal swab, oropharyngeal swab	Panbio COVID-19 Ag Rapid Test Device, immunochro-matography	RT-PCR	NA	NA
Van der Moeren N [20] 2021	Prospective	COVID-19 test center	Symptomatic	4.8	(354/351)	NA	NA	Nose/throat swabs	BD Veritor System for Rapid Detection of SARS-CoV-2	RT-PCR	NA	NA
Peña M [21] 2021	Prospective	Public testing sites	Asymptomatic	11	(854/842)	36.7 (mean) (±16.5, SD)	NA	Nasopharyngeal swab	STANDARD Q COVID-19 Ag Test	RT-PCR	NA	40
Shah MM [22] 2021	Prospective	Community testing site	Asymptomatic and symptomatic	15.8	(2127/2110) the initial BinaxNOW test	NA	≤7	Nasal swab (self-sampling)	BinaxNOW COVID-19 Ag card	RT-PCR	NA	37
Ford L [23] 2021	Prospective	Two universities	Asymptomatic and symptomatic	NA	(1058/1051) symptomatic: 219 asymptomatic: 832	15–24 (87.9%) ≥25 (12.1%)	NA	Nasal swabs	Sofia SARS Antigen, fluorescent immunoassay	RT-PCR	NA	40
Berger A [24] 2021	Prospective	Community-based testing centers	Asymptomatic and symptomatic	NA	(1064/1064)	34 (±12.5, SD)	NA	Nasopharyngeal swab	Panbio COVID-19 Ag Rapid Test Device, Standard Q COVID-19 Ag kit,	RT-PCR	21.5 (14.2–34.2)	NA
Stokes W [25] 2021	Prospective	Community COVID-19 assessment centers	Symptomatic	NA	(1641/1641)	39 (5–90)	NA	Nasopharyngeal swab	Panbio COVID-19 Ag Rapid Test Device, immunochro-matography	RT-PCR	22.1 (13.2–33.9), (E-gene)	35
Takeuchi Y [26] 2021	Prospective	Drive-through-type at a PCR center	Asymptomatic and symptomatic	NA	(1186/1186)	23	2	Nasopharyngeal swab	QuickNavi™-COVID19 Ag	RT-PCR	NA	NA
Gili A [27] 2021	Prospective	Schools, prisons, elderly care homes, and from hospital healthcare worker surveillance programs	NA	5.2	(1738/1738)	NA	NA	Nasopharyngeal swab	Lumipulse^®^ SARS-CoV-2 antigen assay	RT-PCR	NA	35
Pollock NR [28] 2021	Prospective	Drive-through testing site	Asymptomatic and symptomatic	12.7	(2308/2308)	all ages	7	Anterior nasal swab	BinaxNOW COVID-19 Ag card	RT-PCR	26.9 (for adults, asymptomatic) 20.5 (for adults, symptomatic for ≤7 days)	40
Okoye NC [29] 2021	Prospective	A university setting	Asymptomatic	1.7	(2645/2638)	24 (mean) (15–86)	NA	Nasal swab	BinaxNOW COVID-19 Ag card	RT-PCR	17.6	NA
Prince-Guerra JL [30] 2021	Prospective	Two community-based testing sites	Asymptomatic and symptomatic	8.7	(3419/3419)	41 (10–95)	4 (0–210)	Anterior nasal swab	BinaxNOW COVID-19 Ag card	RT-PCR	22 (symptomatic) 22.5 (asymptomatic)	NA
Iglὁi Z 2021 [31]	Prospective	Drive-through testing location	Asymptomatic and symptomatic	19.2	(3615/970) symptomatic: 886	42 (18–86)	4	Nasopharyngeal swab	STANDARD Q COVID-19 Ag Test	RT-PCR	23.6 (15.6–37.4), (E-gene)	NA
Landaas ET [32] 2021	Prospective	test station	Asymptomatic and symptomatic	6.3	(4025/3991)	≥10	NA	Throat/nasopharyngeal swabs	Panbio COVID-19 Ag Rapid Test Device, immunochro-matography	RT-PCR	24.5 (symptomatic) 28.2 (asymptomatic)	NA
Pilarowski G [33] 2020	Prospective	at a plaza	Participants in an urban commercial transport hub	7.2	(3302/3302)	<13, 13–18, >18	NA	Nasal swab	BinaxNOW™ COVID-19 Ag Card	RT-PCR	NA	35

COVID-19 = coronavirus disease 2019; CT = cycle threshold; NA = not available; RT-PCR = reverse-transcription polymerase chain reaction; SD = standard deviation.

**Table 2:** Statistical data of included studies.

Study	True Positive	False Positive	False Negative	True Negative
Pollock NR [16] 2021	135	21	99	1243
García-Fiñana M [17] 2021	28	3	42	5431
Lindner AK [18] 2021	33	0	7	104
Krüger LJ [19] 2021	92	1	14	1001
Van der Moeren N [20] 2021	16	0	1	334
Peña M [21] 2021	51	3	22	766
Shah MM [22] 2021	258	7	76	1769
Ford L [23] 2021	37	15	17	982
Berger A [24] 2021	276	1	39	748
Stokes W [25] 2021	231	2	37	1371
Takeuchi Y [26] 2021	91	0	14	1081
Gili A [27] 2021	90	86	0	1562
Pollock NR [28] 2021	226	12	66	2004
Okoye NC [29] 2021	24	0	21	2593
Prince-Guerra JL [30] 2021	157	4	142	3116
Iglὁi Z [31] 2021	158	4	28	780
Landaas ET [32] 2021	186	3	64	3738
Pilarowski G [33] 2020	201	13	3	3085

**Table 3:** Quality of studies.

Study	Risk of Bias				Applicability Concerns		
	Patient Selection	Index Test	Reference Standard	Flow and Timing	Patient Selection	Index Test	Reference Standard
Pollock NR [16] 2021	L	L	L	H	L	L	L
García-Fiñana M [17] 2021	L	L	L	H	L	L	L
Lindner AK [18] 2021	L	L	L	H	L	L	L
Krüger LJ [19] 2021	L	L	L	H	L	L	L
Van der Moeren N [20] 2021	U	L	L	H	L	L	L
Peña M [21] 2021	U	L	L	H	L	L	L
Shah MM [22] 2021	U	L	L	H	L	L	L
Ford L [23] 2021	U	L	L	H	L	L	L
Berger A [24] 2021	U	L	L	L	L	L	L
Stokes W [25] 2021	U	L	L	H	L	L	L
Takeuchi Y [26] 2021	U	L	L	L	H	L	L
Gili A [27] 2021	U	L	L	L	L	L	L
Pollock NR [28] 2021	L	L	L	L	L	L	L
Okoye NC [29] 2021	U	L	L	H	L	L	L
Prince-Guerra JL [30] 2021	L	L	L	H	L	L	L
Iglὁi Z [31] 2021	L	L	L	H	L	L	L
Landaas ET [32] 2021	U	L	L	H	L	L	L
Pilarowski G [33] 2020	L	L	L	L	L	L	L

H = high risk of bias; L = low risk of bias; U = unclear risk of bias.

## Data Availability

All data generated or analyzed during this study are included in this published article (and its Supplementary Information files).

## Supplementary Materials

The following are available online at https://www.mdpi.com/article/10.3390/ijerph182111451/s1, Figure S1: Sensitivities and specificities of included studies. Table S1: Search strategy. Table S2: Statistical data of subgroup analyses.
