# Supplementary material for: Diagnostic Accuracy of SARS-CoV-2 Antigen Tests for Community Transmission Screening: A Systematic Review and Meta-Analysis
Source: Int J Environ Res Public Health. 2021 Oct 30;18(21):11451. doi: 10.3390/ijerph182111451 (PMC8583375; doi:10.3390/ijerph182111451)
Supplement: Supplementary file 1 [file ijerph-18-11451-s001.zip › Figure S1_Sensitivities and specificities.pdf]

Supplementary Material

Sensitivities and specificities of included studies

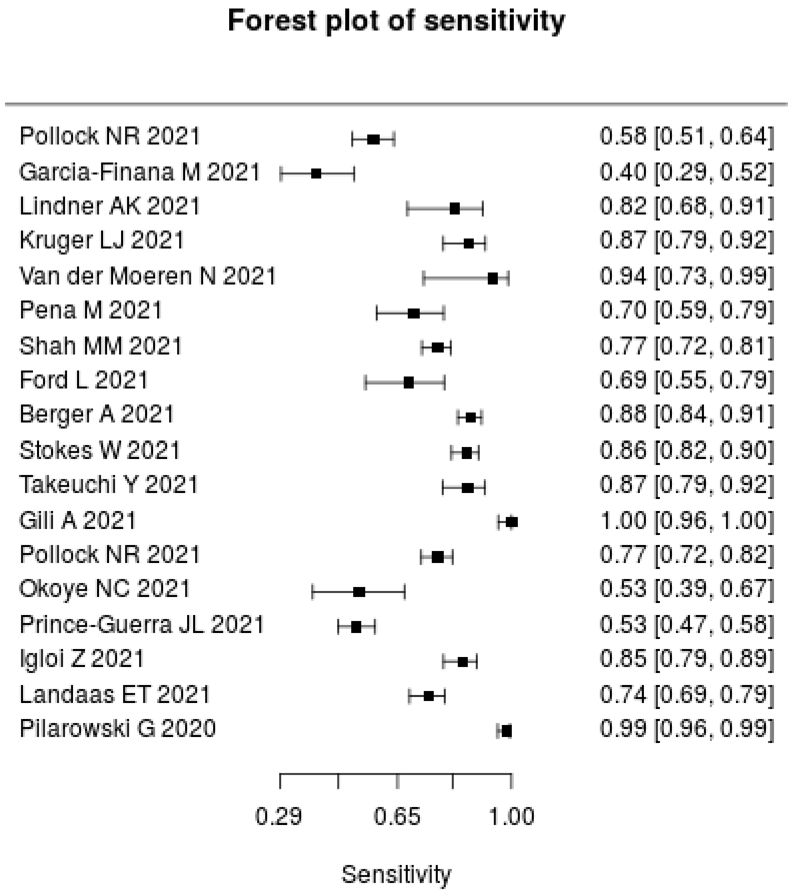

Sensitivities and 95% CIs of antigen tests for SARS-CoV-2 from the included studies

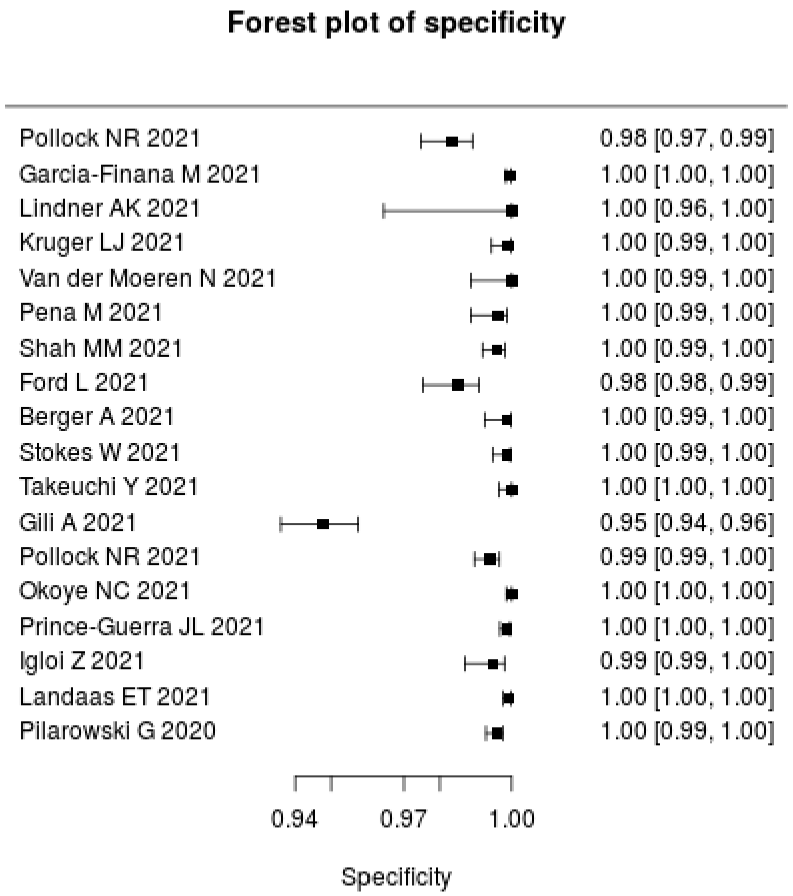

Specificities and 95% CIs of antigen tests for SARS-CoV-2 from the included studies
