# Supplementary material for: Diagnostic Accuracy of SARS-CoV-2 Antigen Tests for Community Transmission Screening: A Systematic Review and Meta-Analysis
Source: Int J Environ Res Public Health. 2021 Oct 30;18(21):11451. doi: 10.3390/ijerph182111451 (PMC8583375; doi:10.3390/ijerph182111451)
Supplement: Supplementary file 1 [file ijerph-18-11451-s001.zip › Table S1_Search strategy_0801.pdf]

Supplementary Material

Search strategy

Primary outcomes: pooled sensitivity and specificity of antigen tests for SARS-CoV-2

**Draft search strategy for each electronic database queried: PubMed, Embase, Cochrane Library, and Biomed Central.** For each search listed below, no start date was applied, and databases were searched from their inception or date of the earliest available publication.

Database	PubMed (including MEDLINE) 407 results
**Description**	Search performed considering terms by "SARS-CoV-2 and antigen test/ RT-PCR and sensitivity" and terms controlled by Medical Subjects Headings (MeSH)
**1#** ("covid 19"[All Fields] OR "covid 19"[MeSH Terms] OR "covid 19 vaccines"[All Fields] OR "covid 19 vaccines"[MeSH Terms] OR "covid 19 serotherapy"[All Fields] OR "covid 19 serotherapy"[Supplementary Concept] OR "covid 19 nucleic acid testing"[All Fields] OR "covid 19 nucleic acid testing"[MeSH Terms] OR "covid 19 serological testing"[All Fields] OR "covid 19 serological testing"[MeSH Terms] OR "covid 19 testing"[All Fields] OR "covid 19 testing"[MeSH Terms] OR "sars cov 2"[All Fields] OR "sars cov 2"[MeSH Terms] OR "severe acute respiratory syndrome coronavirus 2"[All Fields] OR "ncov"[All Fields] OR "2019 ncov"[All Fields] OR (("coronavirus"[MeSH Terms] OR "coronavirus"[All Fields] OR "cov"[All Fields]) AND 2019/11/01:3000/12/31[Date - Publication]) OR ("sars cov 2"[MeSH Terms] OR "sars cov 2"[All Fields] OR "severe acute respiratory syndrome coronavirus 2"[All Fields]) OR ("sars cov 2"[MeSH Terms] OR "sars cov 2"[All Fields] OR "sars cov 2"[All Fields])) AND ((("antigen s"[All Fields] OR "antigene"[All Fields] OR "antigenes"[All Fields] OR "antigenic"[All Fields] OR "antigenically"[All Fields] OR "antigenicities"[All Fields] OR "antigenicity"[All Fields] OR "antigenized"[All Fields] OR "antigens"[MeSH Terms] OR "antigens"[All Fields] OR "antigen"[All Fields])	

AND ("research design"[MeSH Terms] OR ("research"[All Fields] AND "design"[All Fields]) OR "research design"[All Fields] OR "test"[All Fields])) OR (("sars cov 2"[MeSH Terms] OR "sars cov 2"[All Fields] OR "sars cov 2"[All Fields]) AND ("antigen s"[All Fields] OR "antigene"[All Fields] OR "antigenes"[All Fields] OR "antigenic"[All Fields] OR "antigenically"[All Fields] OR "antigenicities"[All Fields] OR "antigenicity"[All Fields] OR "antigenized"[All Fields] OR "antigens"[MeSH Terms] OR "antigens"[All Fields] OR "antigen"[All Fields])) OR ("mass screening"[MeSH Terms] OR ("mass"[All Fields] AND "screening"[All Fields]) OR "mass screening"[All Fields]) OR ("community participation"[MeSH Terms] OR ("community"[All Fields] AND "participation"[All Fields]) OR "community participation"[All Fields]))
**2#** ("reverse transcriptase polymerase chain reaction"[MeSH Terms] OR ("reverse"[All Fields] AND "transcriptase"[All Fields] AND "polymerase"[All Fields] AND "chain"[All Fields] AND "reaction"[All Fields]) OR "reverse transcriptase polymerase chain reaction"[All Fields] OR ("rt"[All Fields] AND "pcr"[All Fields]) OR "rt pcr"[All Fields] OR ("reverse transcriptase polymerase chain reaction"[MeSH Terms] OR ("reverse"[All Fields] AND "transcriptase"[All Fields] AND "polymerase"[All Fields] AND "chain"[All Fields] AND "reaction"[All Fields]) OR "reverse transcriptase polymerase chain reaction"[All Fields]) OR ("covid 19 nucleic acid testing"[MeSH Terms] OR ("covid 19"[All Fields] AND "nucleic"[All Fields] AND "acid"[All Fields] AND "testing"[All Fields]) OR "covid 19 nucleic acid testing"[All Fields] OR "covid 19 nucleic acid testing"[All Fields])) AND ("hypersensitivity"[MeSH Terms] OR "hypersensitivity"[All Fields] OR "sensitive"[All Fields] OR "sensitively"[All Fields] OR "sensitives"[All Fields] OR "sensitivities"[All Fields] OR "sensitivity and specificity"[MeSH Terms] OR ("sensitivity"[All Fields] AND "specificity"[All Fields]) OR "sensitivity and specificity"[All Fields] OR "sensitivity"[All Fields] OR ("sensitivity and specificity"[MeSH Terms] OR ("sensitivity"[All Fields] AND "specificity"[All Fields]) OR "sensitivity and specificity"[All Fields] OR "specificity"[All Fields] OR "specific"[All Fields] OR "specifically"[All Fields] OR "specification"[All Fields] OR "specifications"[All Fields] OR "specificities"[All Fields] OR "specifics"[All Fields] OR "specifities"[All Fields] OR "specify"[All Fields]))
**(1# AND 2#)**

Advanced search: ("covid 19"[All Fields] OR "covid 19"[MeSH Terms] OR "covid 19 vaccines"[All Fields] OR "covid 19 vaccines"[MeSH Terms] OR "covid 19 serotherapy"[All Fields] OR "covid 19 serotherapy"[Supplementary Concept] OR "covid 19 nucleic acid testing"[All Fields] OR "covid 19 nucleic acid testing"[MeSH Terms] OR "covid 19 serological testing"[All Fields] OR "covid 19 serological testing"[MeSH Terms] OR "covid 19 testing"[All Fields] OR "covid 19 testing"[MeSH Terms] OR "sars cov 2"[All Fields] OR "sars cov 2"[MeSH Terms] OR "severe acute respiratory syndrome coronavirus 2"[All Fields] OR "ncov"[All Fields] OR "2019 ncov"[All Fields] OR (("coronavirus"[MeSH Terms] OR "coronavirus"[All Fields] OR "cov"[All Fields]) AND 2019/11/01:3000/12/31[Date - Publication]) OR ("sars cov 2"[MeSH Terms] OR "sars cov 2"[All Fields] OR "severe acute respiratory syndrome coronavirus 2"[All Fields]) OR ("sars cov 2"[MeSH Terms] OR "sars cov 2"[All Fields] OR "sars cov 2"[All Fields])) AND ((("antigen s"[All Fields] OR "antigene"[All Fields] OR "antigenes"[All Fields] OR "antigenic"[All Fields] OR "antigenically"[All Fields] OR "antigenicities"[All Fields] OR "antigenicity"[All Fields] OR "antigenized"[All Fields] OR "antigens"[MeSH Terms] OR "antigens"[All Fields] OR "antigen"[All Fields]) AND ("research design"[MeSH Terms] OR ("research"[All Fields] AND "design"[All Fields]) OR "research design"[All Fields] OR "test"[All Fields])) OR (("sars cov 2"[MeSH Terms] OR "sars cov 2"[All Fields] OR "sars cov 2"[All Fields]) AND ("antigen s"[All Fields] OR "antigene"[All Fields] OR "antigenes"[All Fields] OR "antigenic"[All Fields] OR "antigenically"[All Fields] OR "antigenicities"[All Fields] OR "antigenicity"[All Fields] OR "antigenized"[All Fields] OR "antigens"[MeSH Terms] OR "antigens"[All Fields] OR "antigen"[All Fields])) OR ("mass screening"[MeSH Terms] OR ("mass"[All Fields] AND "screening"[All Fields]) OR "mass screening"[All Fields]) OR ("community participation"[MeSH Terms] OR ("community"[All Fields] AND "participation"[All Fields]) OR "community participation"[All Fields])) AND (("reverse transcriptase polymerase chain reaction"[MeSH Terms] OR ("reverse"[All Fields] AND "transcriptase"[All Fields] AND "polymerase"[All Fields] AND "chain"[All Fields] AND "reaction"[All Fields]) OR "reverse transcriptase polymerase chain reaction"[All Fields] OR ("rt"[All Fields] AND "pcr"[All Fields]) OR "rt pcr"[All Fields] OR ("reverse transcriptase polymerase chain reaction"[MeSH Terms] OR ("reverse"[All Fields] AND "transcriptase"[All Fields] AND "polymerase"[All Fields] AND "chain"[All Fields] AND "reaction"[All

Fields]) OR "reverse transcriptase polymerase chain reaction"[All Fields]) OR ("covid 19 nucleic acid testing"[MeSH Terms] OR ("covid 19"[All Fields] AND "nucleic"[All Fields] AND "acid"[All Fields] AND "testing"[All Fields]) OR "covid 19 nucleic acid testing"[All Fields] OR "covid 19 nucleic acid testing"[All Fields])) AND ("hypersensitivity"[MeSH Terms] OR "hypersensitivity"[All Fields] OR "sensitive"[All Fields] OR "sensitively"[All Fields] OR "sensitives"[All Fields] OR "sensitivities"[All Fields] OR "sensitivity and specificity"[MeSH Terms] OR ("sensitivity"[All Fields] AND "specificity"[All Fields]) OR "sensitivity and specificity"[All Fields] OR "sensitivity"[All Fields] OR ("sensitivity and specificity"[MeSH Terms] OR ("sensitivity"[All Fields] AND "specificity"[All Fields]) OR "sensitivity and specificity"[All Fields] OR "specificity"[All Fields] OR "specific"[All Fields] OR "specifically"[All Fields] OR "specification"[All Fields] OR "specifications"[All Fields] OR "specificities"[All Fields] OR "specifics"[All Fields] OR "specifities"[All Fields] OR "specifity"[All Fields])))

**Database**	**Embase 366 results**
**Description**	Search performed considering terms by "SARS-CoV-2 and antigen test/ RT-PCR and sensitivity" and terms controlled by Medical Subjects Headings (MeSH)
**1#**(('covid 19' OR severe) AND acute AND respiratory AND syndrome AND coronavirus AND 2 OR 'sars cov 2') AND (((antigen AND test OR 'sars cov 2') AND antigens OR mass) AND screening OR community) AND participation	
**2#** (('rt pcr' OR reverse) AND transcriptase AND polymerase AND chain AND reaction OR 'covid 19') AND nucleic AND acid AND testing AND (sensitivity OR specificity)	
**(1# AND 2#)**	
**Advanced search:** (('covid 19' OR severe) AND acute AND respiratory AND syndrome AND coronavirus AND 2 OR 'sars cov 2') AND (((antigen AND test OR 'sars cov 2') AND antigens OR mass) AND screening OR community) AND participation AND (('rt pcr' OR reverse) AND transcriptase AND polymerase AND chain AND reaction OR 'covid 19') AND nucleic AND acid AND testing AND (sensitivity OR specificity)	

Database	**Cochrane Library 23 results**
**Description**	Search performed considering terms by "SARS-CoV-2 and antigen test/ RT-PCR and sensitivity" and terms controlled by Medical Subjects Headings (MeSH)
**1#** (COVID-19 OR severe acute respiratory syndrome coronavirus 2 OR SARS-CoV-2) AND (antigen test OR SARS-COV-2 antigens OR Mass Screening OR Community Participation)	
**2#** (RT-PCR OR Reverse Transcriptase Polymerase Chain Reaction OR COVID-19 Nucleic Acid Testing) AND (sensitivity OR specificity)	
**#1 and #2**	
**Basic search: 23 results**	

Database	**BioMed Central 2 results**
**Description**	Search performed considering terms by "SARS-CoV-2 and antigen test/ RT-PCR and sensitivity" and terms controlled by Medical Subjects Headings (MeSH)
**1#** (COVID-19 OR severe acute respiratory syndrome coronavirus 2 OR SARS-CoV-2) AND (antigen test OR SARS-COV-2 antigens OR Mass Screening OR Community Participation)	
**2#** (RT-PCR OR Reverse Transcriptase Polymerase Chain Reaction OR COVID-19 Nucleic Acid Testing) AND (sensitivity OR specificity)	
**#1 and #2**	
**Basic search: 2 results**	
