# Supplementary material for: Diagnostic Accuracy of SARS-CoV-2 Antigen Tests for Community Transmission Screening: A Systematic Review and Meta-Analysis
Source: Int J Environ Res Public Health. 2021 Oct 30;18(21):11451. doi: 10.3390/ijerph182111451 (PMC8583375; doi:10.3390/ijerph182111451)
Supplement: Supplementary file 1 [file ijerph-18-11451-s001.zip › Table S2_Subgroup analyses.pdf]

Supplementary Material

Statistical data of subgroup analyses

Statistical data of symptomatic participants in the community

Study	True Positive	False Positive	False Negative	True Negative
Pollock NR 2021	52	9	17	163
Lindner AK 2021	33	0	7	104
Krüger LJ 2021	79	1	12	620
Van der Moeren N 2021	16	0	1	334
Shah MM 2021	221	2	60	905
Ford L 2021	30	1	8	180
Takeuchi Y 2021	66	0	6	699
Pollock NR 2021	116	1	12	376
Prince-Guerra JL 2021	113	0	63	651
Pilarowski G 2020	120	5	0	546

Statistical data of asymptomatic participants in the community

Study	True Positive	False Positive	False Negative	True Negative
Pollock NR 2021	83	12	82	1080
García-Fiñana M 2021	28	3	42	5431
Krüger LJ 2021	12	0	2	374
Peña M 2021	51	3	22	766
Shah MM 2021	33	5	15	824
Ford L 2021	7	14	9	802
Pollock NR 2021	110	11	54	1628
Okoye NC 2021	24	0	21	2593
Prince-Guerra JL 2021	44	4	79	2465

Statistical data of participants within 7 days after symptom onset

Study	True Positive	False Positive	False Negative	True Negative
Shah MM 2021	199	2	44	684
Pollock NR 2021	104	0	7	335
Pilarowski G 2020	120	5	0	546

Statistical data of participants with Ct value less than or equal to 35

Study	True Positive	False Positive	False Negative	True Negative
García-Fiñana M 2021	28	3	42	5431
Ford L 2021	37	15	11	988
Stokes W 2021	231	2	37	1371
Gili A 2021	90	86	0	1562
Pilarowski G 2020	201	13	3	3085

Statistical data of participants less than or equal to 18 of ages

Study	True Positive	False Positive	False Negative	True Negative
Pollock NR 2021	26	7	20	200
Pollock NR 2021	94	7	41	786
Pilarowski G 2020	28	2	1	178
